# New national outcome data on fresh versus cryopreserved donor oocytes

**DOI:** 10.1186/s13048-017-0378-4

**Published:** 2018-01-05

**Authors:** Vitaly A. Kushnir, Sarah K. Darmon, David H. Barad, Norbert Gleicher

**Affiliations:** 1 The Center for Human Reproduction, 21 East 69th Street, New York, NY 10021 USA; 2 Department of Obstetrics and Gynecology, Wake Forest School of Medicine, Winston-Salem, NC USA; 3 Foundation for Reproductive Medicine, New York, NY USA; 4 Department of Obstetrics and Gynecology, University of Vienna School of Medicine, Vienna, Austria; 5 Stem Cell Biology and Molecular Embryology Laboratory, The Rockefeller University, New York, NY USA

**Keywords:** In vitro fertilization, Oocyte donation, Cryopreservation, Vitrification

## Abstract

**Background:**

Improvements in oocyte cryopreservation techniques and establishment of cryopreserved donor oocyte banks have led to improved access to and lower cost of donor oocytes, upending the traditional practice of fresh oocyte donation. The objective of this study was to examine national trends in utilization and live birth rates with fresh versus cryopreserved donor oocytes.

**Methods:**

A retrospective analysis of 2013 through 2015 aggregate U.S. national data reported by the Society for Assisted Reproductive Technology which included 30,160 IVF cycles with either fresh or cryopreserved donor oocytes was performed.

**Results:**

During the study period utilization of fresh oocyte donations rapidly declined by 32.9%, while cryopreserved oocyte donation increased by 44.4%. Fresh donor oocytes produced significantly higher live birth rates per recipient cycle start than cryopreserved donor oocytes (51.1% vs. 39.7%). Over the three-year study period fresh donor oocytes produced stable live birth rates per recipient cycle start while those with cryopreserved oocytes significantly declined year-by-year.

**Conclusion:**

Despite rising popularity of cryopreserved donor oocytes, prospective patients should be counselled that fresh donor oocytes still represent standard of care due to higher live birth rates.

## Background

Improved survival of human oocytes following cryopreservation via the vitrification technique prompted a change in the position of the American Society for Reproductive Medicine (ASRM) on the experimental nature of oocyte cryopreservation in 2013 [1]. Though ASRM recommended against routine donor oocyte banking until more robust clinical data on safety and efficacy of cryopreserved donor oocytes were generated, multiple commercial cryopreserved oocyte donor banks were nevertheless concurrently established in the U.S. Based on 2013 U.S. national data, we previously reported that live birth rates for recipients of donated cryopreserved oocytes were lower than those of fresh oocytes [2]. This conclusion was questioned by colleagues who argued that, as fertility centers gain expertise with oocyte cryopreservation and thawing, success rates would align with those of fresh oocytes [3]. To test this hypothesis, we here report two additional years of national outcome data that have become available since our original publication, demonstrating that the par between fresh and cryopreserved oocyte over these 2 years has actually significantly widened.

## Methods

We compared utilization and live birth rates of fresh versus cryopreserved oocyte donation cycles in 2013–2015 publicly available reports of the Society for Assisted Reproductive Technology (SART) [4]. These reports are based on anonymized aggregate data of U.S. in vitro fertilization (IVF) centers, which collectively perform over 90% of all IVF cycles. These source data undergo annual validation as previously described [5]. Because this study investigated only anonymized data, it received expedited Institutional Review Board approval. Cycles involving cryopreserved embryos were excluded.

Live birth rates were compared using the two-tailed Fisher’s exact test and Wilson confidence interval for binomial proportions. A Poisson regression model was used to analyze live birth rates overall trends. A *P*-value of <0.05 was considered statistically significant. All statistical analyses were performed using SAS version 9.4 statistical software.

## Results

Table 1 demonstrates that during the study period the utilization of fresh oocyte donations rapidly declined by 32.9%; (*P* < 0.0001), while cryopreserved oocyte donation rapidly increased by 44.4%; (*P* < 0.0001). Concurrently, cycle cancellation rates with fresh oocytes declined from 11.7 to 9.1% (*P* < 0.0001), while those with use of cryopreserved oocytes increased from 8.5 to 15.0% (*P* < 0.0001). The table also shows that the number of embryos transferred over the study period was similar in both study groups.

**Table 1:** Fresh and Cryopreserved Donor Oocyte cycles reported to Society for Assisted Reproductive Technology, 2013–2015

	Fresh Donor Oocytes			Cryopreserved Donor Oocytes		
Year(s)	2013	2014	2015^a^	2013	2014	2015^a^
Number of cycles	8921	6929	5982	2227	2886	3215
Average number of transferred embryos	1.7	1.6	1.6	1.6	1.6	1.6
Cancelled Cycles (%)	11.7	7.0	9.1	8.5	12.4	15.0

^a^2015 data were calculated from a preliminary report distributed by SART

**Fig. 1:**
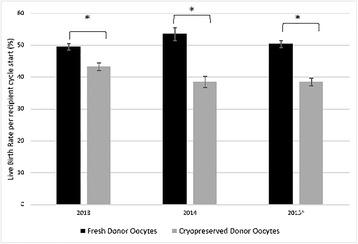
Live birth rates with fresh versus cryopreserved donated oocytes, 2013–2015. * *P* < 0.001; Live birth rates were compared using the two-tailed Fisher’s exact test and Wilson confidence interval for binomial proportions. ^^^ 2015 data were calculated from a preliminary report distributed by SART

Despite similar numbers of transferred embryos, live birth rates over the whole study period were higher with fresh than cryopreserved oocytes per recipient cycle start (51.1% vs. 39.7%, *P* < 0.0001) and per embryo transfer (56.4% vs. 45.3%, P < 0.0001). Figure 1 demonstrates that live birth rates per recipient cycle start were higher with fresh donor oocytes in each of the studied years. Moreover, fresh donor oocytes produced stable live birth rates per recipient cycle start over the 3 years (*P* = 0.2925), while those with cryopreserved oocytes declined year-by-year (*P* = 0.0094).

Restricting the analysis to patients with a primary diagnosis of uterine factor infertility revealed a total of only 151 cycles with fresh oocytes and 54 cycles with cryopreserved oocytes over the three-year study period. Cycle cancellation rates were 13.2% and 13%, while live birth rates per cycle start were 49.7% and 37.0% with fresh and cryopreserved oocytes respectively.

Analyzing outcomes only for cycles with elective single embryo transfer (eSET), live birth rates per embryo transfer were 53.7% with fresh vs. 46.5% with cryopreserved oocytes (*P* < 0.0001). Absolute and relative differences in live birth rates were, thus, smaller with eSET. While percentages of pregnancies resulting in term births were similar for eSET cycles with fresh and cryopreserved donor oocytes (84.6% vs. 83.1%).

## Discussion and conclusions

Despite lower and, seemingly, over time further declining live birth rates with cryopreserved oocytes, here presented data demonstrate that oocyte donation in the U.S. is undergoing a rapid transition from fresh to cryopreserved oocytes. This trend is likely driven by improved access to, and lower cost of cryopreserved oocytes, and facilitated by the rapid establishment of cryopreserved donor oocyte banks [6].

This analysis of aggregate outcome data did not allow adjustments for confounding patient characteristics which would require access to patient level data. Based on 2013 patient level national data, a recent report by investigators from the Centers for Disease Control and Prevention, where such adjustments were performed, also reported decreased likelihood of pregnancy and live birth per started cycle with cryopreserved donor oocytes but no difference when cycles were restricted to those proceeding to embryo transfer [7]. We did attempt to account for patients diagnosed with uterine factor infertility but found that there were relatively few patients with this diagnosis, and they had similar cancellation and live birth rates as other patients using fresh and cryopreserved donor oocyte respectively.

Considering rising cancellation as well as declining live birth rates over the study period in cryopreserved oocyte donation cycles, here presented data not only confirm but actually strengthen our prior reports that suggested significant outcome differences in favor of fresh oocytes [2, 6].

With increasing use of cryopreserved oocytes, live birth rates did not, as had been suggested [3], improve with spreading practice from early centers of excellence to the general IVF community, − but declined. This is not only confirmed by declining live birth rates with cryopreserved oocytes but also by increasing cycle cancellation rates. Since cycle cancellations are rare in egg donation cycles, this observation strengthens the conclusion that, in comparison to fresh donor oocytes, infertile women will experience significantly lower pregnancy and live birth chances with utilization of cryopreserved oocytes. This observation contradicts much smaller scale studies, which served as original justification for the ASRM’s lifting of the experimental designation of oocyte cryopreservation [1].

Reasons for these increasing differences in live birth rates have yet to be determined. There are many potential explanations to consider: For example, it is possible that cryopreservation and subsequent thawing, negatively affects an oocyte’s and resulting embryo’s pregnancy potential. Though oocyte cryopreservation, with switch from slow-freezing to vitrification over the last decade, has greatly improved [8, 9], the latter is a more complex technique to master, as is the thawing process for so cryopreserved oocytes. Oocyte donor selection by commercial donor oocyte banks may not be as rigid as donor selection for fresh donor cycles by infertility centers. Finally, it is also possible that observed differences stem from smaller allotments of cryopreserved than fresh oocytes per recipient, a practice which reduces cycle costs by sharing oocytes from a single donor cycle among several recipients.

The SART data set did not permit analysis of cumulative live birth rates, which include the initial IVF cycle plus any subsequent transfers of surplus cryopreserved embryos. In our clinical experience these rates are even more in favor of fresh oocyte donation since patients utilizing cryopreserved donor oocytes typically have fewer surplus embryos available following the initial cycle. Moreover, performance of surplus cryopreserved embryos derived from previously cryopreserved oocytes has not been well studied, though initial reports appear reassuring [10].

Use of cryopreserved donated oocytes offers advantages over fresh oocytes, including simplified logistics of coordinating donor and recipient cycles, access to a broader pool of donors particularly for ethnic minorities, the ability to ship frozen oocytes over long distances, thereby reducing the need for reproductive tourism and, potentially, lower costs per treatment cycle [6]. However, as demonstrated in this report, there are also distinct disadvantages to use of cryopreserved oocytes, including rising cycle cancellation rates likely a reflection of oocyte thaw survival, lower live birth rates, availability of only small batches of oocytes, which preclude future pregnancies of genetic siblings, and potentially higher costs per live birth.

Fresh oocyte donation must, therefore, still be considered the ‘gold standard’ in oocyte donation, and patients should be accordingly counselled. In this context it is also important to note that, according to ASRM recommendations, routine donor oocyte banking should not be recommended unless clinical data on safety and equivalent efficacy of oocyte cryopreservation become available [1].
